# An Integrated Model to Improve Medication Reconciliation in Oncology: Prospective Interventional Study

**DOI:** 10.2196/31321

**Published:** 2021-12-20

**Authors:** Alessandro Passardi, Patrizia Serra, Caterina Donati, Federica Fiori, Sabrina Prati, Roberto Vespignani, Gabriele Taglioni, Patrizia Farfaneti Ghetti, Giovanni Martinelli, Oriana Nanni, Mattia Altini, Giovanni Luca Frassineti, Martina Vittoria Minguzzi

**Affiliations:** 1 Department of Medical Oncology IRCCS Istituto Romagnolo per lo Studio dei Tumori (IRST) “Dino Amadori” Meldola Italy; 2 Unit of Biostatistics and Clinical Trials IRCCS Istituto Romagnolo per lo Studio dei Tumori (IRST) “Dino Amadori” Meldola Italy; 3 Oncology Pharmacy Unit IRCCS Istituto Romagnolo per lo Studio dei Tumori (IRST) “Dino Amadori” Meldola Italy; 4 Oncology Unit IRCCS Istituto Romagnolo per lo Studio dei Tumori (IRST) “Dino Amadori” Meldola Italy; 5 IT Service IRCCS Istituto Romagnolo per lo Studio dei Tumori (IRST) “Dino Amadori” Meldola Italy; 6 A.S.SO.FARM Community Pharmacy Ravenna Italy; 7 Federfarma Community Pharmacy Rimini Italy; 8 Hematology Unit IRCCS Istituto Romagnolo per lo Studio dei Tumori (IRST) “Dino Amadori” Meldola Italy; 9 Healthcare Administration Azienda Unità Sanitaria Locale (AUSL) Romagna Ravenna Italy; 10 Healthcare Administration IRCCS Istituto Romagnolo per lo Studio dei Tumori (IRST) “Dino Amadori” Meldola Italy

**Keywords:** medication recognition, medication reconciliation, IT platform, community pharmacies, healthcare transitions, pharmacy, oncology, drug incompatibility, information technology, drug interactions

## Abstract

**Background:**

Accurate medication reconciliation reduces the risk of drug incompatibilities and adverse events that can occur during transitions in care. Community pharmacies (CPs) are a crucial part of the health care system and could be involved in collecting essential information on conventional and supplementary drugs used at home.

**Objective:**

The aim of this paper was to establish an alliance between our cancer institute, Istituto Romagnolo per lo Studio dei Tumori (IRST), and CPs, the latter entrusted with the completion of a pharmacological recognition survey. We also aimed to integrate the national information technology (IT) platform of CPs with the electronic medical records of IRST.

**Methods:**

Cancer patients undergoing antiblastic treatments were invited to select a CP taking part in the study and to complete the pharmacological recognition step. The information collected by the pharmacist was sent to the electronic medical records of IRST through the new IT platform, after which the oncologist performed the reconciliation process.

**Results:**

A total of 66 CPs completed surveys for 134 patients. An average of 5.9 drugs per patient was used at home, with 12 or more used in the most advanced age groups. Moreover, 60% (80/134) of the patients used nonconventional products or critical foods. Some potential interactions between nonconventional medications and cancer treatments were reported.

**Conclusions:**

In the PROF-1 (Progetto di Rete in Oncologia con le Farmacie di comunità della Romagna) study, an alliance was created between our cancer center and CPs to improve medication reconciliation, and a new integrated IT platform was validated.

**Trial Registration:**

ClinicalTrials.gov NCT04796142; https://clinicaltrials.gov/ct2/show/NCT04796142

## Introduction

Medication reconciliation is the process of drawing up a complete and accurate list of all medications being taken by an individual patient, including drug name, dosage, frequency, and route of administration, and comparing them with the medications listed in the patient’s medical records or medication prescriptions. The aim of reconciliation is to reduce the risk of errors of omission, duplication, incorrect doses or timing, and adverse drug-drug or drug-disease interactions [1-3]. The Joint Commission on Accreditation of Health Care Organizations added the concept of reconciliation across the continuum of care as a national patient safety goal [4]. It has also been defined as one of the best strategies for maintaining the quality of care by the Agency for Healthcare Research and Quality [5] and is one of the 5 elements in the “High 5s Project” launched by the World Health Organization [6].

Reconciliation must be periodically performed at both the hospital and territorial level [7] and at every transition of care [8], especially when new medications are prescribed or rewritten as several professionals may be involved. The efficacy and quality of this process depends mainly on a preliminary pharmacological survey (recognition step), which not only takes into account the medications prescribed to the patient, but also the phytotherapeutic and homeopathic products, supplements, and foods taken, as well as any habit that might negatively affect patient safety [5]. The success of the recognition step depends on the interlocutor's ability to promote within the patient a sense of empowerment and responsibility in relation to treatment adherence [9]. The use of innovative drugs for the treatment of cancer is constantly increasing [10], and many are unknown to the vast majority of nonexperts in the field. This increases the risk of pharmacological discrepancies in the medications prescribed (or self-prescribed) in the interval between each access to the cancer center caring for the patient [11-13]. In particular, nonconventional medicinal and health preparations such as over-the-counter products, herbal medicines, and supplements are sources of potential pharmacological discrepancy and should be closely monitored [5]. Cancer patients frequently resort to such remedies in an attempt to improve their quality of life, often compromised by the side effects of the treatments, or to help them cope with the emotional aspects related to living with cancer.

Therapy errors have economic implications. The World Health Organization has estimated the cost of therapy errors to be at US $42 billion annually and has set a goal of reducing these costs (which are attributed to weaknesses in health care systems) by 50% within the next 5 years [14]. In the area of oncology, the extent of the economic risk is increased by the burden of expected outcomes and by the high costs of anticancer treatments for the UK National Health Service. In 2018, in Italy, these costs were estimated to be at €5659 million (US $6360 million), constituting the first item of public health expenditure and exceeding that of cardiovascular drugs [15].

Community pharmacies (CPs) are a crucial part of the health care system. They are neighborhood health care facilities whose activities include dispensing medications, treating minor ailments, and offering advice on well-being. CP staff also frequently build up a close relationship with their clients, and they are highly knowledgeable about food supplements as they are responsible for around 80% of the sales of these products on the market [16]. It is therefore relatively easy for CPs to gather information to obtain a complete and accurate picture of the use of conventional drugs and supplements at home.

The territory of Romagna has a population of 1,281,243, spread over 3 provinces (Forlì-Cesena, Ravenna, and Rimini), and represents 28.6% (366,436/1,281,243) of the Emilia-Romagna region in northern Italy. There are 356 CPs in Romagna. Our cancer institute (IRCCS Istituto Romagnolo per lo Studio dei Tumori [IRST], “Dino Amadori”) is also based in this area. The center has a high level of computerization and standardization of all therapeutic, clinical, and experimental processes, guaranteeing maximum safety for patients and full traceability of the actions carried out by each professional, clinician, pharmacist, and nurse during their daily activities. The national association representing private CPs (Federfarma) recently developed an information technology (IT) platform (Dottorfarma) in collaboration with an e-commerce company (Promofarma) that can be used by CPs nationwide.

Given these premises, an alliance created between IRST and the CPs of Romagna seemed to provide the perfect opportunity [17] to improve the pharmacological reconciliation process and to bridge the “pharmaceutical gap” between the health care system and the patients. We carried out a prospective, interventional, nonpharmacological study of the first phase of the network project in oncology with the community pharmacies of Romagna (Progetto di Rete in Oncologia con le Farmacie di comunità della Romagna [PROF-1]) on a new model for medication reconciliation. Our overall aims were to create an organizational model and IT platform, assess their ability to promote the shared management of therapies by patients, pharmacists, and health care professionals (thus improving adherence to treatment and the home management of side effects), and evaluate the acceptability of this model by patients.

## Methods

The team of investigators included the IRST oncology group (oncologists, hospital pharmacists, and nurses), IRST and Federfarma IT Services, CPs, and scientific representatives of private and public pharmacy associations. The study team also worked closely with lawyers from Federfarma and Promofarma to take care of the delicate aspects of professional responsibility and data privacy. During the feasibility analysis of the project (PROF-1 trial), the basic network model was defined by jointly identifying the individual professionals and their respective responsibilities. The investigators agreed that each of the professionals would be responsible for the accuracy of the information provided, while the medication reconciliation process would be the exclusive responsibility of physicians. This decision meant that the data transmitted from a CP to IRST would only be valid after the clinician had downloaded, integrated, and confirmed the information. Regarding the CPs’ task of collecting patient data, it was agreed that the pharmacist transmitting data directly to IRST medical records would not be able to view or modify the entered information.

A drug recognition form was created and validated in a pilot study carried out on breast cancer patients [18]. The form was divided into 5 sections (medications, critical foods, supplements, phytotherapeutic products, and homeopathic products). Information would be gathered for each compound (eg, active ingredient and trade name; pharmaceutical form and route of administration; dosage, posology, start or end of intake, reason for intake, and prescriber). Initially conceived as a paper document, the form was digitized in both the Dottorfarma IT platform and IRST medical records for the purposes of the trial.

A total of 200 patients were considered for the PROF-1 study. A formal sample size calculation for this prospective study was not performed due to the lack of preliminary data and the exploratory intent of the study. Eligible patients were required to meet the following criteria: being adults ≥18 years old, of either gender; having an Eastern Cooperative Oncology Group performance status of ≤1; undergoing anticancer treatment; having a clear understanding of the Italian language; and granting written informed consent.

**Figure 1:**
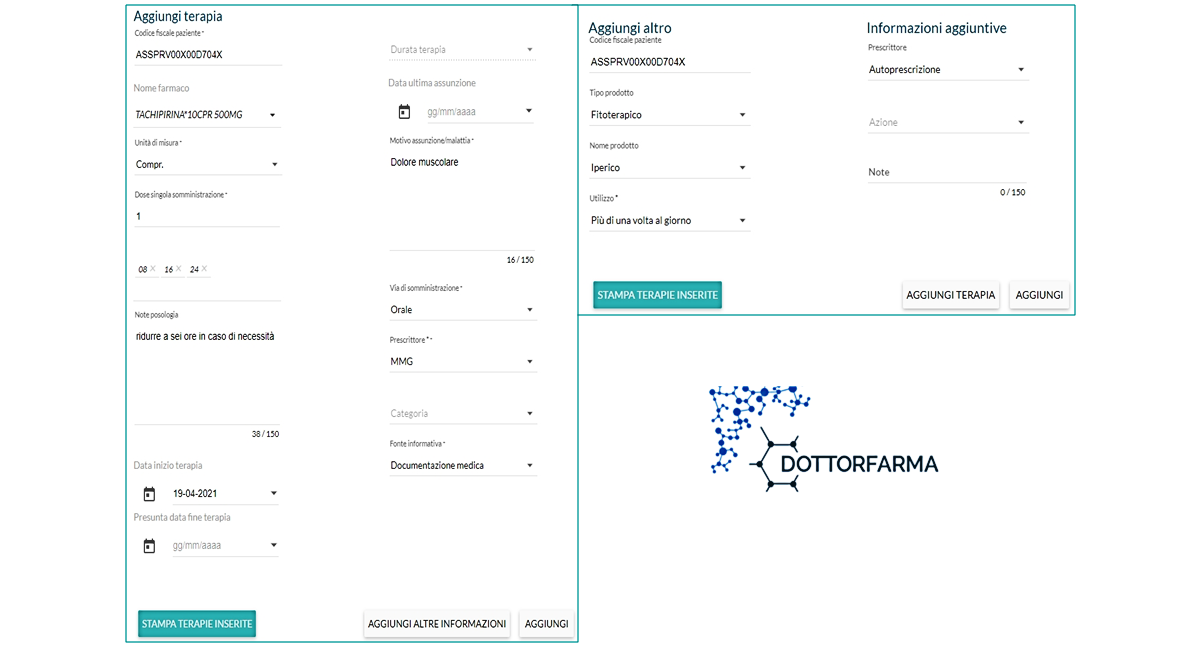
Dottorfarma electronic scheme of the recognition form.

**Figure 2:**
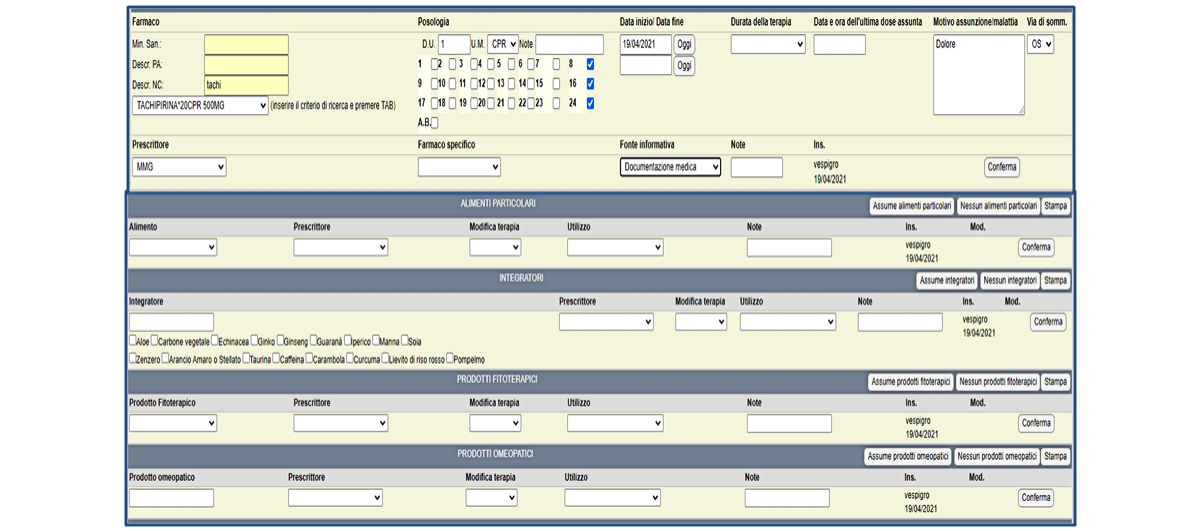
Drug history section of the IRST (Istituto Romagnolo per lo Studio dei Tumori) electronic medical records.

When the patients came to the institute for anticancer treatment, health care professionals provided information about the trial and obtained written informed consent from those willing to take part. The patients were asked to choose one of the CPs accredited for the trial and to book an appointment for the pharmacological recognition step. The accredited pharmacist, after ensuring that the patient had provided written informed consent and completed a privacy form, interviewed the patient in a private consultation area, completing the online drug recognition form in the Dottorfarma IT platform (Figure 1), which has the same format as that of IRST electronic medical records. The pharmacist took into account the information provided by the patient or delegated caregiver and that retrieved from drug packages or other products used by the patient at home (and brought to the interview), paper referrals, or prescriptions from specialists. The recognition data were sent to IRST through the Dottorfarma IT platform and automatically saved in a specific medical history file in the electronic medical records (Figure 2). Upon confirmation of the next course of chemotherapy, the oncologist downloaded the pharmacological recognition form and performed the reconciliation process. For the purposes of the present trial, the processes of recognition and reconciliation were carried out only once for each enrolled patient. Following the completion of the drug recognition and reconciliation processes, the patients were asked to complete a satisfaction questionnaire.

Descriptive statistical analyses were performed on all case series (absolute and relative frequency for categorical variables and median and quantiles for continuous variables). All statistical analyses were carried out using SAS Software, version 9.4 (SAS Institute).

This study was reviewed and approved by the AUSL (Azienda unità sanitaria locale) Romagna/IRST Ethics Committee (approval number 1722, October 26, 2016) and was conducted in accordance with the ethical standards laid down in the 1964 Declaration of Helsinki and later versions. The participants provided written informed consent to take part in the study.

The data sets generated and analyzed during the current study are available from the corresponding author upon reasonable request.

## Results

A dialog between IRST and local CP associations was opened, leading to the creation of a specific agreement between the 2 parties. The first step involved the implementation of the Dottorfarma IT platform, initially with a one-way communication flow. The pharmacological recognition form was digitized on both the Dottorfarma IT platform and IRST electronic medical records, and the 2 systems were synchronized.

### Pharmacy Recruitment and Training

Following an open invitation, out of 365, 120 (34%) of the public and private CPs of Romagna initially agreed to take part in the study. Pharmacy managers or a representative from each CP took part in a brief training course organized by IRST to inform the participants about the recognition and reconciliation process, to present the PROF-1 study, and to provide instructions on how to complete the pharmacological recognition form. A total of 108 CPs (84 private and 24 public CPs) finally agreed to participate in the trial (36 in the province of Forlì-Cesena, 26 in the province of Rimini, and 46 in the province of Ravenna). The enrolled patients chose 66 of these to complete the pharmacological recognition survey (Multimedia Appendix 1).

### Patients

From April 2017 to April 2018, 200 patients were enrolled onto the trial. Among these, 137 (68.5%) underwent the pharmacological recognition interview as planned, while 63 (31.5%) were not evaluable, 39 (19.5%) withdrew consent, 11 (5.5%) were excluded for technical problems, and 13 (6.5%) spontaneously dropped out) (Multimedia Appendix 1). Patient characteristics are shown in Table 1.

The majority of the patients were female (83, 61%), with a median age of 63 years Most patients lived in the province of Forlì-Cesena, where our institute is based. The majority were undergoing chemotherapy (alone or in combination with targeted therapies), mainly administered intravenously. The list of anticancer drugs administered to the enrolled patients is reported in Table 2.

The analysis of the data entered by CPs revealed a total of 805 medications declared by the patients, with an average of 5.9 drugs per patient taken at home. As many as 12 or more medications were being taken by 6 patients over the age of 65 years. Drug distribution, identified in the recognition form according to the Anatomical Therapeutic Chemical classification, is reported in Table 3 (1st and 2nd levels).

Of the total 805 declared drugs, 29.4% (n=237) and 20.7% (n=167) were used to treat problems in the gastrointestinal and cardiovascular systems, respectively. The former drugs were mainly used to contrast chemotherapy side effects and cancer-related symptoms (eg, proton pump inhibitors and antacids, antiemetics, laxatives, and antidiarrheals), while the latter mainly included antihypertensive drugs and statins. Drugs for the central nervous system represented 13.2% (n=106) of all drugs and comprised mainly analgesics and antidepressants or anxiolytics. Hormones (55, 6.8%), drugs for musculoskeletal disorders (47, 5.8%), antithrombotic or antianemic drugs (43, 5.3%) and systemic antimicrobials (32, 4%) were also being taken. Although the recognition of anticancer drugs was not required (this information was already present in the medical records), some patients reported oral anticancer drugs in their list of medicines. Further interesting information was obtained from the recognition form; ie, 87.6% (n=705) of drugs were taken orally. Moreover, sources of information varied, coming from medical documents (145, 18%), drug lists (227, 28.2%), product packages (86, 10.7%), patient declarations (270, 33.5%), and caregiver declarations (73, 9.1%). The main prescribers were specialist physicians (542, 67.3%) and general practitioners (204, 25.3%), while 1.5% (n=12) of the patients were self-prescribers.

**Table 1:** Patient characteristics (n=137).

Variables		Values
Age (years), range (years)		67 (39-85)
**Gender, n (%)**		
	Male	54 (39)
	Female	83 (61)
**Provence of residence, n (%)**		
	Forlì-Cesena	86 (63)
	Ravenna	36 (26)
	Rimini	8 (6)
	Other	7 (5)
**Site of disease** **, n (%)**		
	Gastrointestinal tract	36 (26)
	Breast	31 (23)
	Genitourinary tract	27 (20)
	Hematologic malignancy	17 (12)
	Lung	14 (10)
**Anticancer treatment** **, n (%)**		
	Chemotherapy	55 (39)
	Chemotherapy plus targeted therapy	28 (20)
	Targeted therapy	21 (16)
	Immunotherapy	13 (10)
	Hormonal therapy	20 (15)
**Administration route, n (%)**		
	Intravenous	74 (54)
	Intravenous and oral	33 (24)
	Oral	30 (22)

**Table 2:** Anticancer drugs (n=462).

Category and name of anticancer drugs		Values
**Cytotoxic drugs, n (%)**		
	Paclitaxel	38 (8.2)
	5-fluorouracil	36 (7.8)
	Capecitabine	36 (7.8)
	Cyclophosphamide	31 (6.7)
	Gemcitabine	30 (6.4)
	Oxaliplatin	29 (6.3)
	Carboplatin	25 (5.4)
	Cisplatin	23 (5.0)
	Irinotecan	18 (3.9)
	Docetaxel	17 (3.7)
	Vinorelbine	14 (3.0)
	Doxorubicin	13 (2.8)
	Nab^a^-paclitaxel	11 (2.4)
	Liposomal doxorubicin	10 (2.2)
	Epirubicin	9 (1.9)
	Bendamustine	8 (1.7)
	Eribulin	7 (1.5)
	Etoposide	7 (1.5)
	Melfalan	7 (1.5)
	Trifluridine+tipiracil	6 (1.3)
	Pemetrexed	5 (1.1)
	Total	380 (82.2)
**Targeted therapy n, (%)**		
	Trastuzumab	14 (3.0)
	Rituximab	11 (2.4)
	Bevacizumab	11 (2.4)
	Cetuximab	6 (1.3)
	Regorafenib	7 (1.5)
	Total	49 (10.6)
**Immunotherapy n, (%)**		
	Nivolumab	13 (2.9)
**Hormonal therapy n, (%)**		
	Fulvestrant	9 (1.9)
	Abiraterone acetate	6 (1.3)
	Enzalutamide	5 (1.1)
	Total	20 (4.3)

^a^Nab: nanoparticle albumin-bound.

**Table 3:** Distribution of drugs (n=805) detected in the recognition (Anatomical Therapeutic Chemical, 1st and 2nd levels).

ATC^a^ classification		Values
**Gastrointestinal tract and metabolism, n (%)**		
	Proton pump inhibitors and antacids	82 (10.2)
	Antiemetics and prokinetics	33 (4.1)
	Vitamin A and D and associations	30 (3.7)
	Insulin and hypoglycemic agents	24 (3.0)
	Laxatives	18 (2.2)
	Antidiarrheal and anti-inflammatory intestinal	17 (2.1)
	Mineral supplements	14 (1.7)
	Other	19 (2.3)
	Total	237 (29.4)
**Cardiovascular system, n (%)**		
	Antihypertensives (including diuretics)	128 (16.0)
	Statins	34 (4.2)
	Other	5 (0.6)
	Total	167 (20.7)
**Central nervous system, n (%)**		
	Analgesics (including opioids)	53 (6.6)
	Antidepressants, anxiolytics, and sedatives	36 (4.5)
	Other	17 (2.1)
	Total	106 (13.2)
**Hormones (excluding insulin and sex hormones), n (%)**		
	Systemic corticosteroids	42 (5.2)
	Thyroid preparations	12 (1.5)
	Other	1 (0.1)
	Total	55 (6.8)
**Antineoplastic agents and immunomodulators, n (%)**		
	Oral anticancer drugs	39 (4.8)
	Intramuscular hormonal antagonists	11 (1.4)
	Total	50 (6.2)
**Musculoskeletal system, n (%)**		
	Antigout drugs	23 (2.8)
	Anti-inflammatory drugs (including NSAIDs^b^)	16 (2.0)
	Bisphosphonates	6 (0.7)
	Other	2 (0.3)
	Total	47 (5.8)
**Blood and hematopoietic organs, n (%)**		
	Antithrombotic drugs	32 (4.0)
	Antianemic drugs	11 (1.3)
	Total	43 (5.3)
**Systemic antimicrobials, n (%)**		
	Antibacterials	22 (2.7)
	Antivirals	9 (1.1)
	Antifungals	1 (0.1)
	Total	32 (4.0)
Genitourinary tract and sex hormones, n (%)		20 (2.5)
Respiratory system, n (%)		18 (2.2)
Skin, n (%)		7 (1.0)
Other, n (%)		23 (2.9)

^a^ATC: Anatomical Therapeutic Chemical.

^b^NSAIDs: nonsteroidal anti-inflammatory drugs.

In addition, of a total of 137 patients, 83 (60.5%) reported an intake of 201 nonconventional medications (supplements, phytotherapeutics, or homeopathic products), and 39 (28.5%) reported having taken foods considered critical for the potential interactions with medications (Table 4). A detailed analysis of the recognition form revealed that the most widely represented critical substances were coffee (23 patients, 16.8%), green tea (11 patients, 8%), aloe (6 patients, 4.4%), turmeric (5 patients, 3.6%), fermented red rice (5 patients, 3.6%), ginger (3 patients, 2.2%) and manna (3 patients, 2.2%).

An evaluation of drug-drug interaction was not carried out as it was not one of the aims of this paper. However, we investigated the potential for interaction between nonconventional products and cancer treatments (Table 4), identifying 2 possible interactions with aloe vera, 3 with manna, 1 with echinacea, 1 with ginseng, 2 with ginger, and 3 with red yeast rice. After analyzing the components present in the supplements taken by the patients but not present in our list of critical compounds, the following possible interactions emerged: 2 interactions with milk thistle, 2 with berberine, 1 with alpha lipoic acid, 1 with vitamin C, 1 with folic acid, and 1 with *Cordyceps sinensis*.

A total of 106 patients completed and returned the satisfaction questionnaire. Of these, 77 (72%) considered the reconciliation process as very important, 74 (70%) thought that the involvement of the pharmacist was very useful, and 87 (82%) reported no difficulty in going to the chosen pharmacy to complete the pharmacological recognition survey.

**Table 4:** Critical foods, nonconventional products, and potential interactions with cancer treatments.

Product		Value	Potential interaction
**Critical foods, n (%)**			
	Coffee	23 (16.8)	—^a^
	Green tea	11 (8)	—
	Black tea	—	—
	Bitter orange	—	—
	Carom	—	—
	Grapefruit	1 (0.7)	Regorafenib
	Chili pepper	—	—
	Pepper	—	—
	Turmeric	5 (3.6)	Doxorubicin, cyclophosphamide
**Phytotherapeutic, n (%)**			
	Aloe vera	6 (4.4)	Paclitaxel, docetaxel
	Charcoal	—	—
	Echinacea	1 (0.7)	Dexamethasone
	Ginseng	1 (0.7)	Etoposide
	Guarana	—	—
	Hypericum	—	—
	Red yeast rice	5 (3.6)	Cyclophosphamide, paclitaxel, etoposide
	Manna	3 (2.2)	Capecitabine, enzalutamide, abiraterone
	Soy	—	—
	Ginger	3 (2.2)	5-fluorouracil, capecitabine
**Other compounds^b^, n (%)**			
	Milk thistle	2 (1.5)	Regorafenib, paclitaxel
	Alpha-lipoic acid	1 (0.7)	Cisplatin
	Vitamin C	1 (0.7)	Doxorubicin
	Folic acid	1 (0.7)	Capecitabine
	Berberine	2 (1.5)	Trastuzumab, vinorelbine
	*Cordyceps sinensis*	1 (0.7)	Dexamethasone

^a^Not applicable.

^b^Not reported in the official list of critical compounds, but with possible critical interactions in the post hoc analysis.

## Discussion

### Principal Findings

The PROF-1 trial achieved its goal of creating a new model for medication recognition and reconciliation processes in oncology thanks to the close cooperation between our institute and CPs, the implementation of an integrated IT platform, and the active participation of cancer patients.

An interest in being actively involved in the setting up of a new hospital territory network was clearly demonstrated by both public and private CPs in their willingness to take part, at no cost, in the project. A total of 120 CPs participated in the training course, and 108 agreed to enrol patients, the latter process involving a commitment of around 30 minutes for each survey to integrate into routine pharmacy activities. To the best of our knowledge, this was the first trial addressing the problem of medication reconciliation to include private entities not directly involved in the care of cancer patients.

PROF-1 confirmed the high number of medications used by patients at home (an average of 5.9 drugs per patient), especially in the most advanced age groups. Even more striking was the evidence of an increasing number of patients who used nonconventional products or critical foods. These results underline the importance of pharmacological recognition and reconciliation processes, which, despite being mandatory ministerial measures [1,3], are often neglected by oncologists because of the pressures of daily clinical activity. An alliance with CPs could thus lead to a significant improvement in the situation [19-21]. Numerous attempts have been made to improve the medication reconciliation process, including pharmacist-related interventions and IT platforms, with interesting results in terms of reduction in medication discrepancies and potential adverse events [22]. However, none of the proposed models included the presence of community pharmacists.

Our study highlighted some limitations in this new model, the first concerning patient empowerment. Although the patients were personally involved and expressed a high degree of satisfaction through the questionnaire, a certain number who signed the informed consent did not go to the chosen CP to complete the recognition step. This may have been due to a deterioration in their clinical conditions or to the side effects of chemotherapy, given that the study population was recruited among those undergoing antiblastic therapy, regardless of prognosis or type of treatment. This is an important aspect to bear in mind as it suggests that some patients may not be suitable for this type of project. A review of the communication channels between patient and CP, including the use of the telephone or email rather than direct access, could perhaps improve this issue. Another important limitation was that, given the exploratory nature of the trial, only one recognition-reconciliation process was planned for each patient, making it impossible to verify the advantage of a continuous exchange of information from a series of repeated processes to monitor changes in medications taken at home. A new trial (PROF-2) is ongoing to further implement and validate the model, this time incorporating the repetition of the recognition-reconciliation processes before each chemotherapy cycle. Another imitation concerns drug-drug interactions. As stated above, the use of a surprisingly high number of drugs, supplements, and nonconventional products were declared by patients, raising the question of possible interactions. However, we did not focus on the drug-drug interactions that emerged from the reconciliations performed by oncologists, as this was not an aim of the study. Conversely, our analysis of the potential interactions between cancer drugs and nonconventional products confirmed the importance of this issue. Finally, the difficult reproducibility of the proposed model must be emphasized as a possible limitation because of some basic requirements (ie, a high level of computerization of the centers taking part and the strict regulation of data processing and patient privacy). The strengths that made it possible to complete the project were the solidity of the computerized medical records present in our cancer institute, the presence of an IT platform that could be shared by all private and public CPs, and the approval of the project by the local ethics committee after in-depth teamwork to resolve the issues of professional responsibility, data ownership, and privacy management.

Based on the results from this pilot study, the alliance between the cancer center and the CPs of Romagna has led to the creation of a cancer network which, albeit initially established to meet a specific need, could help to systematically establish safety pathways for drugs used at home. This initiative could also contribute to increasing adherence to innovative drugs, essential not only for the success of treatment but also for the sustainability of the national health service of every nation. It could also improve the management of ancillary drugs for the prevention of toxicities, often underestimated or self-managed by patients [23]. The PROF-1 project was conceived and designed some years before the COVID-19 pandemic, which has forced the community to acknowledge the importance of the territory in the management of patients’ needs. Within this context, our model proved highly functional not only in terms of the study’s main aim (pharmacological reconciliation), but also in terms of the successful creation of a hospital community network whose impact may well exceed what was originally hypothesized.

### Conclusions

The PROF-1 trial represents an important step forward in medication reconciliation in oncology. The alliance established between our cancer institute and local CPs to enhance medication reconciliation in transitions in care led to the creation of an innovative organizational model and the validation of a new integrated IT platform.

## Appendix

Multimedia Appendix 1 PROF-1 (Progetto di Rete in Oncologia con le Farmacie di comunità della Romagna) trial flow chart.

## Abbreviations

AUSL: Azienda unità sanitaria locale
CP: community pharmacy
IRST: Istituto Romagnolo per lo Studio dei Tumori
IT: information technology
PROF: Progetto di Rete in Oncologia con le Farmacie di comunità della Romagna
