# Supplementary figures and images for: An Integrated Model to Improve Medication Reconciliation in Oncology: Prospective Interventional Study
Source: J Med Internet Res. 2021 Dec 20;23(12):e31321. doi: 10.2196/31321 (PMC8726040; doi:10.2196/31321)

**
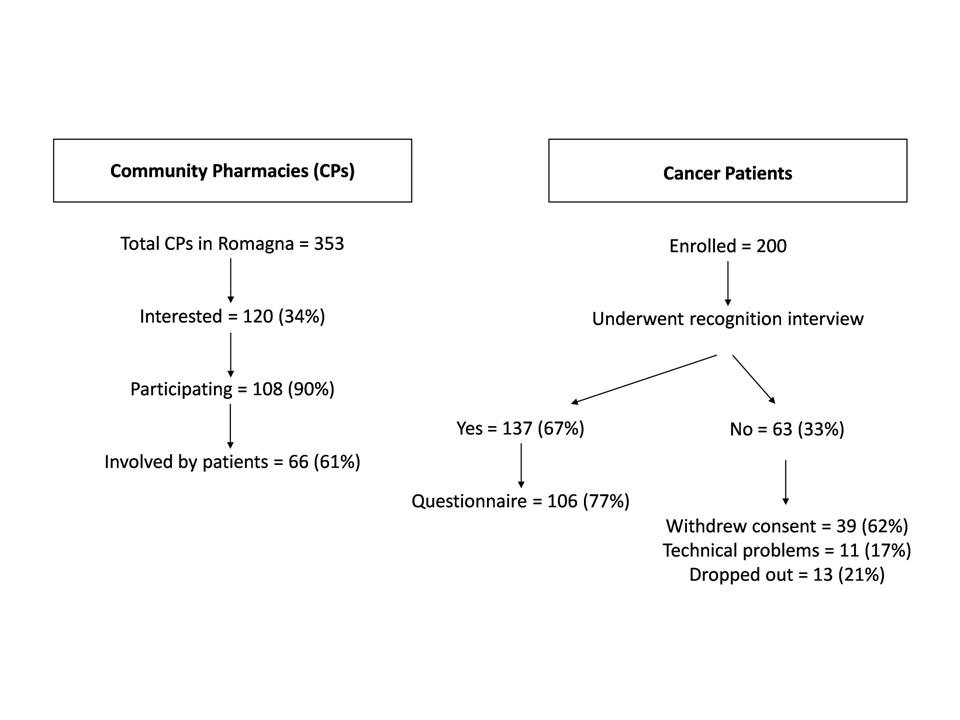
**

**MULTIMEDIA APPENDIX 1**

**PROF-1 Trial flow-chart**

Supplement: Multimedia Appendix 1 [file jmir_v23i12e31321_app1.docx]
